# Interactions comportementales et rythmes d’activité de *Glossina palpalis gambiensis* et *G. tachinoides* (Diptera : Glossinidae) en galerie forestière au Burkina Faso

**DOI:** 10.1051/parasite/2012193217

**Published:** 2012-08-15

**Authors:** E. Salou, J.B. Rayaissé, C. Laveissière, A. Sanon, P. Solano

**Affiliations:** 1 CIRDES URBIO, LAMIVECT 01 BP 454 Bobo-Dioulasso 01 Burkina Faso; 2 380, route de la Virvée 33240 Saint-Romain-la-Virvée France; 3 Université de Ouagadougou, Laboratoire d’Entomologie fondamentale et appliquée, UFR SVT Ouagadougou Burkina Faso .; 4 IRD, UMR INTERTRYP IRD-CIRAD, LAMIVECT, CIRDES 01 BP 454 Bobo-Dioulasso 01 Burkina Faso

**Keywords:** tsé-tsé, galerie, compétition, hauteur de vol, proline, Burkina Faso, tsetse, forest gallery, competition, flight height, proline, Burkina Faso

## Abstract

*Glossina palpalis gambiensis* et *G. tachinoides* sont des vecteurs majeurs des trypanosomoses humaines et animales en Afrique de l’Ouest. Sur une partie de leur aire de répartition, elles sont présentes en sympatrie, mais très peu d’informations sont disponibles sur leurs interactions. Nous avons capturé ces deux espèces en utilisant un système attractif composé d’écrans de tissu noir/bleu/ noir muni de film adhésif, afin de retenir toutes les glossines posées et de pouvoir mesurer la hauteur à laquelle elles se sont posées, ainsi que leur rythme d’activité en fonction de l’heure de la journée. L’étude a eu lieu dans deux zones du sud du Burkina Faso : Kartasso en amont du fleuve Mouhoun, où seule *G. p. gambiensis* est présente, et Folonzo sur le fleuve Comoé, où les deux espèces cohabitent. Les résultats, sur 3 800 glossines capturées, montrent une forte prédominance des captures de *G. tachinoides* par rapport à *G. p. gambiensis* à Folonzo (84 % contre 16 % des captures respectivement). À Kartasso, où elle est seule, *G. p. gambiensis* est capturée en moyenne à 46 cm du sol. À Folonzo, *G. p. gambiensis* est en moyenne attrapée à une hauteur de 65 cm, et *G. tachinoides* à 55 cm, ces différences de hauteurs étant significatives. Les femelles sont capturées en général plus haut que les mâles. Les deux espèces montrent un rythme d’activité similaire en fonction de l’heure de capture, mais seule *G. p. gambiensis* réduit sa hauteur de vol aux heures les plus chaudes. Plusieurs hypothèses, non exclusives, sont évoquées pour expliquer ces hauteurs de capture différentes : la nature de la galerie forestière, un comportement d’approche qui différerait entre espèces, mais aussi la possibilité de phénomènes de compétition interspécifique en relation avec l’utilisation de ressources énergétiques limitées (métabolisme lié à la proline). Sont également discutées les conséquences possibles de ces différences de comportement sur les méthodes de lutte, par exemple lors de l’utilisation d’attractifs olfactifs qui pourraient avoir des efficacités distinctes en fonction de la hauteur de vol.

## Abstract (en)

*Glossina palpalis gambiensis* and *G. tachinoides* are the main vectors of human and animal trypanosomoses in West Africa. In some parts of their distribution area, they co-exist in sympatry, but little is known about their interactions. This study aimed to explore their respective flight height and daily activity when co-existing or alone. Attractive targets were used, made of a black/blue/black cloth covered with adhesive film, so that all tsetse that landed were caught. The study was conducted in two areas in South Burkina Faso: Kartasso, upstream the Mouhoun river, where *G. p. gambiensis* is the only tsetse occurring; and Folonzo, on the Comoé river, where both species occur. Out of more than 3,800 tsetses caught in total, in Folonzo, *G. tachinoides* occurred at higher densities than *G. p. gambiensis* (84 *vs* 16 % of the total densities). The mean height of capture was 55 cm for *G. tachinoides*, and 65 cm for *G. p. gambiensis*. As a comparison, in Kartasso where *G. p. gambiensis* is alone, the mean height of capture was 46 cm, these differences being statistically significant. In average, females were caught higher in altitude than males, and the two species showed a similar activity profile in the day. These results are discussed in the light of differences in the nature of the forest gallery, or possible interspecies competition behaviour in relation with their limited energy metabolism and flight capacities, or also with species differences in landing behavior, linked to host feeding detection. These observations have consequences on control tools releasing attractive odours, which may have contrasted efficacy depending of the flight height of the species.

## Introduction

La mouche tsé-tsé ou glossine, diptère dont les deux sexes sont hématophages, est le vecteur principal de la Trypanosomose Humaine Africaine (THA) ou maladie du sommeil, et des Trypanosomoses Animales Africaines (TAA). Malgré les différentes campagnes de lutte menées dans différentes zones d’Afrique (Cuisance *et al.*, 1984a ; Oladunmade *et al.*, 1985 ; Takken *et al.*, 1986), il apparaît que cette affection parasitaire représente toujours pour les pays touchés un lourd fardeau, sur la santé humaine (Simarro *et al.*, 2008) et le développement de l’agriculture et de l’élevage (Budd *et al.*, 1999). Plusieurs méthodes de lutte existent (Cuisance *et al.*, 1994 ; Bouyer *et al.*, 2010). Cependant, en l’absence de nouvelles molécules pour le traitement, au vu de l’accroissement de la résistance aux trypanocides et de l’impossibilité de contrôler le réservoir animal, la lutte contre le vecteur pour rompre le cycle de transmission est une des bases de la stratégie contre la THA et les TAA (Solano *et al.*, 2010), combinée pour la THA avec le diagnostic et le traitement des malades. 31 espèces et sous-espèces de tsé-tsé occupent dix millions de km2 de terres propices à l’agriculture et à l’élevage. Elles sont réparties en trois sous-genres, également appelés “groupes” selon des critères écologiques (préférences d’habitat) et morphologiques (Jordan, 1993). On distingue les groupes *morsitans* (espèces de savane), *palpalis* (espèces riveraines) et *fusca* (espèces de forêt). Cependant, à l’échelle locale, les répartitions géographiques de certaines espèces différentes (voire de groupes différents) se superposent (Gouteux *et al.*, 1981, 1998). Les comportements des différentes espèces ne sont pas les mêmes, par exemple en réponse aux leurres utilisés (visuels, olfactifs). Il est ainsi rapporté que les captures des espèces du groupe *morsitans* sont augmentées d’un facteur dix lorsqu’un attractif de synthèse est ajouté à un leurre visuel, cette augmentation n’étant que d’un facteur deux à quatre pour les glossines du groupe *palpalis* (Torr & Solano, 2010). La réponse à ces attractifs peut cependant varier même au sein d’un groupe, en l’occurrence *palpalis*. Ainsi, l’adjonction de cet attractif olfactif multiplie par quatre à cinq les captures de *G. tachinoides*, mais seulement par deux celles de *G. p. gambiensis* (Rayaisse *et al.*, 2010). Il est aussi connu que beaucoup d’espèces sont attirées par les formes horizontales, alors que *G. palpalis palpalis* en Côte d’Ivoire l’est plutôt, elle, par les formes verticales (Tirados *et al.*, 2011 ; Rayaisse *et al.*, 2011).

Moins connus sont les rapports de compétition entre espèces. En Côte d’Ivoire, une étude rapporte que l’élimination de l’espèce dominante (*G. p. palpalis* en l’occurrence) par piégeage intensif a été immédiatement suivie par une augmentation du nombre de *G. pallicera* et *G. nigrofusca* (Gouteux *et al.*, 1998). De plus, il faut noter que la glossine est l’une des rares espèces d’insectes (Arrese & Soulages, 2011) qui utilise principalement la proline comme source d’énergie pour l’activité de vol (Bursell, 1964, 1981). Or ces réserves protéiques ont un faible pouvoir énergétique, et la température, en accélérant l’oxydation des réserves énergétiques (proline) dans les muscles du vol, est le principal facteur limitant l’activité de vol. Chez *G. m. morsitans* les fréquences des battements d’ailes sont corrélées négativement à l’augmentation de la température (Hargrove, 1980). À cela peut également s’ajouter la spoliation énergétique résultant de la compétition pour la proline entre le trypanosome et la glossine, en cas d’infection (Bursell, 1981). Compte tenu des dépenses énergétiques lors du vol, il est crucial pour la glossine de développer des stratégies pour les réduire. Cela se traduit par des vols courts successifs (Bursell & Taylor, 1980) et une faible hauteur de vol (Laveissière *et al.*, 1987). Cette faible hauteur de vol est à l’origine du développement des leurres visuels, pièges et écrans de dimensions et de formes variables, ou filets moustiquaires imprégnés, toujours installés au ras du sol et jamais à plus d’un mètre de hauteur (Kuzoe & Schofield, 2005 ; Bauer *et al.*, 2006).

La répartition et l’abondance des mouches du groupe *palpalis* sont fortement tributaires du type de la végétation riveraine (Gruvel, 1975 ; Laveissière, 1978 ; Itard, 1986 ; Laveissière *et al.*, 2000 ; Bouyer *et al.*, 2005). Au Burkina Faso, les galeries forestières sont généralement occupées par *G. p. gambiensis* et *G. tachinoides*. Elles peuvent vivre en sympatrie (de la Rocque, 2001), mais très peu d’éléments sont disponibles pour connaître leurs interactions, notamment en termes de compétition.

L’objectif de ce travail est de caractériser d’éventuelles interactions entre deux espèces de glossines grâce à des analyses de paramètres comportementaux, et grâce à un système d’échantillonnage plus représentatif que le piège classiquement utilisé. Plus spécifiquement, nous nous intéressons :

- aux interactions entre espèces. En mettant un accent particulier sur *G. p. gambiensis*, qui s’adapte même à de fortes densités humaines, nous cherchons à mieux connaître son comportement dans deux zones différentes : l’une où elle est seule, l’autre où elle cohabite avec d’autres espèces, et où elle est minoritaire ;
- au cycle journalier, afin de mesurer les horaires d’activité maximale et minimale des glossines vivant en sympatrie ; les données existantes à ce sujet étant anciennes (Challier, 1973, 1982).

## Matériels et Méthodes

### Zones d’étude

• Galerie forestière dégradée (affluent de la Pindia, bassin du fleuve Mouhoun)

Les travaux ont été menés tout d’abord, en avril 2011, sur la Pindia, affluent de l’amont du fleuve Mouhoun près du village de Kartasso située dans la province du Kénédougou à l’Ouest du Burkina Faso (11° 18’ N – 5° 27’ W). La végétation bordant le cours d’eau est fortement dégradée à cause de la pression démographique et des activités culturales. *G. p. gambiensis* est la principale espèce rencontrée, avec quelques rares *G. tachinoides*. Le cours d’eau est fréquenté par les hommes et le bétail qui constituent les principaux hôtes nourriciers, avec quelques varans (*Varanus niloticus*).

• Galerie forestière conservée (le fleuve Comoé)

En début du mois de mai, les activités ont été conduites au niveau de la réserve Comoé-Léraba, à Folonzo (09° 54’ N – 04° 36’ W). L’existence de quatre espèces de tsé-tsé avait été établie (Laveissière *et al.*, 1981 ; Mérot, 1989 ; Amsler *et al.*, 1994 ; Rayaissé *et al.*, 2009) au niveau de cette localité : *G. tachinoides* fortement majoritaire, avec quelques *G. p. gambiensis* et *G. medicorum* (espèce de forêt du groupe *fusca*) dans la forêt riveraine, et *G. m. submorsitans* dans la savane environnante. En dépit de la pression humaine et du braconnage, la faune sauvage se maintient difficilement : quelques guibs harnachés (*Tragelaphus scriptus*), phacochères (*Phacochaerus aethiopicus*), cobes de buffon (*Kobus kob*), cobes de Fassa (*Kobus ellipsyprimnus*), buffles (*Syncerus cafer*), hippopotames (*Hippopotamus amphibus*), crocodiles (*Crocodilus niloticus*) et varans (V*aranus niloticus*), qui constituent les principaux hôtes nourriciers des glossines.

### Système de capture et protocole expérimental

**Figure 1.**
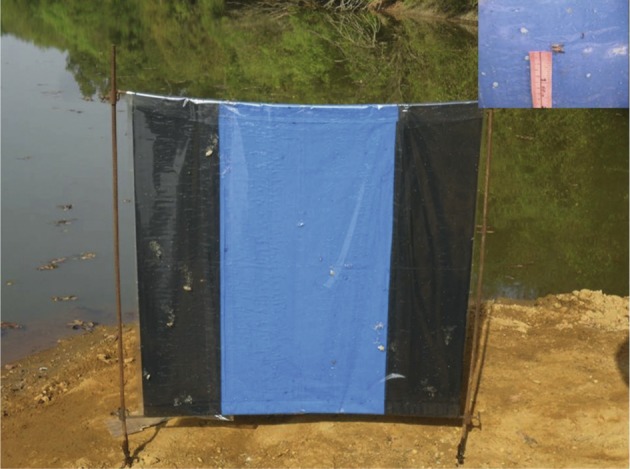
Écran noir/bleu/noir recouvert avec le film adhésif sur la berge du fleuve Comoé. En haut à droite figure un exemple de mesure de la hauteur des glossines capturées.

Les données existantes sur les rythmes d’activité avaient été collectées par captures à la main (Challier, 1973), ou à l’aide de pièges biconiques (Challier *et al.*, 1977 ; Okiwelu, 1982 ; Gouteux & Montery, 1986). Or si le piège est très attractif, nous savons que seulement 20 à 25 % des *G. p. gambiensis* attirées pénètrent effectivement dans la cage (Rayaissé et al., 2010, 2012 ; Esterhuizen *et al.*, 2011). Pour cette expérience, nous avons donc utilisé un écran noir/bleu/noir (Laveissière *et al.*, 1987) de 1 m × 1 m. L’écran est tendu à l’aide de potences métalliques permettant de le recouvrir de film adhésif (Ndegwa & Mihok, 1999 ; Mohamed-Ahmed & Mihok, 2009) qui empêche les glossines de repartir une fois qu’elles se sont posées sur l’écran (figure 1). Ce système a comme avantage supplémentaire de permettre la mesure précise de la hauteur à laquelle les glossines se posent sur le leurre.

Les relevés quotidiens des mouches ont été faits de 8 h 00 à 18 h 00 à des intervalles de deux (2) heures, soit six collectes par jour. Les glossines collectées ont été identifiées par espèce et par sexe. L’écran a été posé sur la berge à une hauteur de 20 cm au dessus du sol, et perpendiculairement au cours d’eau afin de pouvoir intercepter les glossines qui volent. La hauteur de vol a été mesurée sur les deux sites (durant 15 et 20 jours respectivement à Folonzo et Kartasso). À cet effet, la position de chaque glossine posée sur l’écran recouvert de film est mesurée à l’aide d’un ruban gradué (la base de l’écran étant considérée comme l’origine). L’activité s’exprime par le nombre relatif de glossines collectées durant les différentes tranches horaires, ramenées aux captures de la journée (Harley, 1965 ; Gouteux & Monteny, 1986). Elle a été étudiée seulement à Folonzo sur les deux espèces de glossines et pendant 15 jours.

### Analyses statistiques

Pour l’analyse de la hauteur de vol, le modèle linaire généralisé (GLM) a été appliqué, en utilisant l’espèce de la glossine, le sexe et la période de capture comme variables explicatives. La hauteur étant une variable quantitative discrète, les mesures (données brutes) de la position des glossines ont été utilisées pour l’analyse. Pour l’analyse du cycle d’activité, les captures horaires ont été homogénéisées par transformation log10(x+1), puis les moyennes soumises à une ANOVA en utilisant le logiciel R version 2.12.0 (2010–10–15). Pour toutes nos analyses, la valeur maximum du seuil de signification retenu est de 0,05. Du fait de ses faibles effectifs en galerie, *G. m. submorsitans* a été écartée des analyses.

## Résultats

### Espèces de glossines présentes

Sur la Pindia à Kartasso 223 glossines (toutes *G. p. gambiensis*) ont été capturées, dont 86 mâles et 137 femelles. À Folonzo, 3 617 glossines ont été capturées dans les proportions suivantes : 3 035 *G. tachinoides* (84 %), 552 *G. p. gambiensis* (15,2 %) et 30 *G. m. submorsitans* (0,08 %). Le sex-ratio est en faveur des mâles pour chaque espèce capturée : 1,27, 1,53 et 1,72 respectivement.

### Distribution verticale des glossines capturées sur les écrans

• Effet de l’espèce

**Figure 2.**
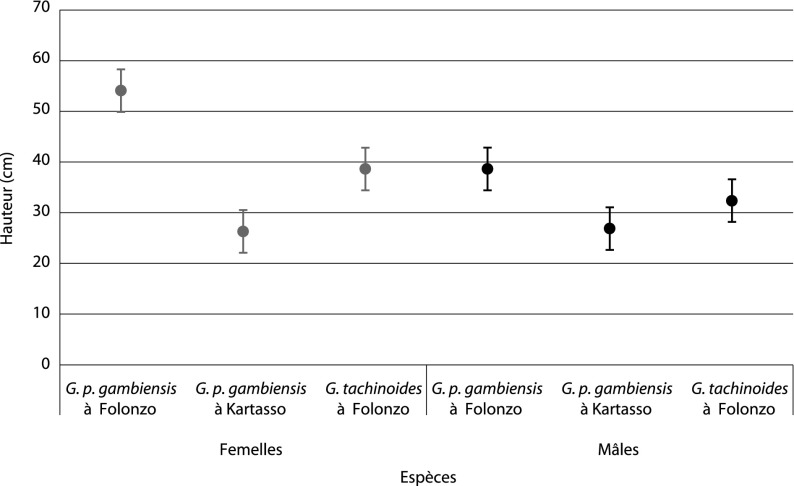
Comparaison de la hauteur de capture selon l’espèce et le sexe. Les chiffres de hauteur représentés ici sont ceux mesurés sur l’écran. Pour avoir la hauteur réelle de vol, il faut ajouter 20 cm qui représentent la hauteur à laquelle la partie basse de l’écran est posée au-dessus du sol.

À Folonzo, la comparaison de la hauteur moyenne entre espèces indique une différence significative : *G. tachinoides* l’espèce majoritaire, est capturée plus bas que *G. p. gambiensis* (respectivement 55,12 cm contre 64,72 cm de hauteur au-dessus du sol, p < 0,004). Ces résultats sont illustrés sur la figure 2, où sont représentées les hauteurs de vol mesurées sur l’écran par espèce et par sexe. À Kartasso, *G. p. gambiensis* vole encore plus près du sol (46,64 cm). La comparaison entre les deux populations allopatriques montre que *G. p. gambiensis* à Folonzo se déplace à une hauteur 1,7 fois plus importante qu’à Kartasso (64,72/46,64, p < 0,001). Il existe aussi une différence significative de hauteur de capture entre *G. tachinoides* à Folonzo (55,12 cm) et *G. p. gambiensis* à Kartasso (46,64 cm) (p < 0,001), qui sont toutes deux dominantes dans leur biotope.

• Effet du sexe

Quelle que soit l’espèce de glossines, les femelles volent significativement plus haut que les mâles (p < 0,0006) à Folonzo. Par contre chez la population de Kartasso, les deux sexes se déplacent pratiquement à la même hauteur (environ 46 cm) (figure 2).

• Hauteur de vol en fonction de l’heure de la journée

**Figure 3.**
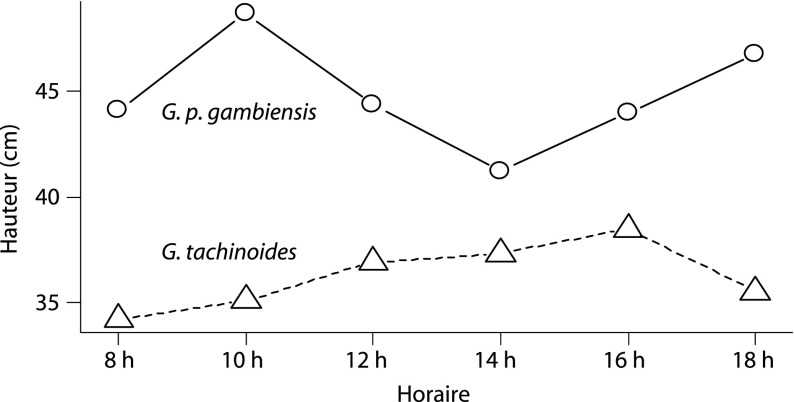
Variations de la hauteur de capture *G. tachinoides* et *G. p. gambiensis* en fonction de l’horaire. La hauteur indique ici la pose de la glossine sur l’écran.

De façon générale, la hauteur moyenne de vol semble varier en fonction du temps au cours de la journée. *G. p. gambiensis* vole plus haut le matin pour atteindre une hauteur maximale (~ 50 cm) à 10 h 00, avant de descendre à un minimum (~ 40 cm) à 14 h 00. Elle vole de nouveau plus haut aux heures plus clémentes. L’allure de la courbe est différente chez *G. tachinoides* : sa hauteur de vol croît de 8 h 00 à 16 h 00, avant de redescendre en fin de journée jusqu’à 18 h 00 (figure 3).

### Rythme circadien d’activité

**Figure 4.**
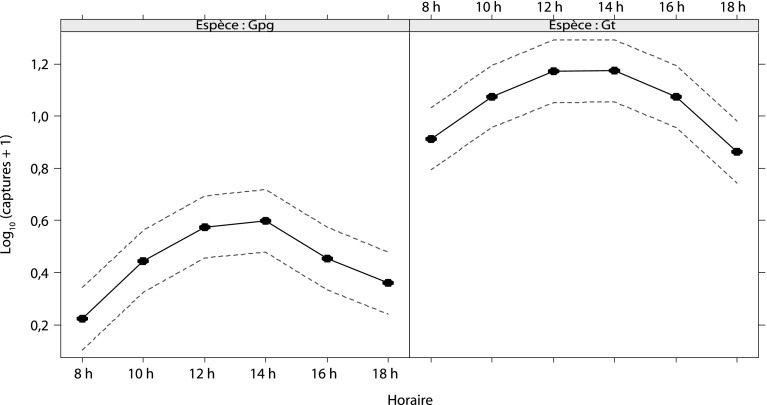
Profils de l’activité journalière de *G. tachinoides* et *G. p. gambiensis* à Folonzo.

**Figure 5.**
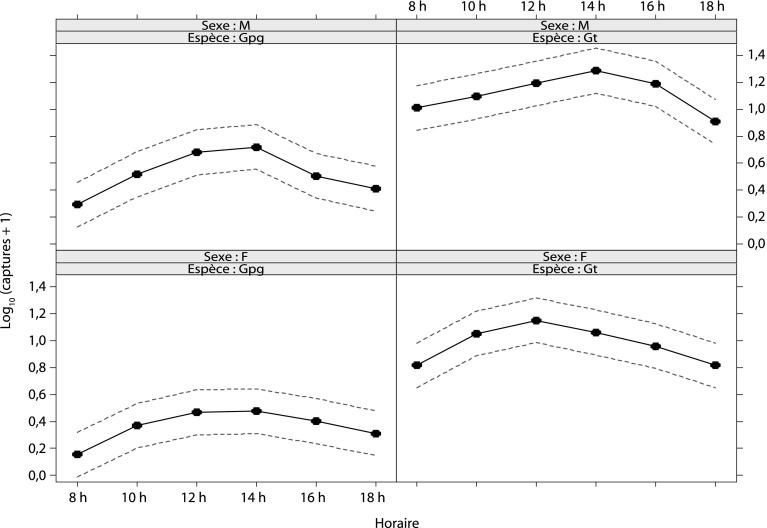
Comparaison du cycle d’ativité journalière des mâles et des femelles de *G. tachinoides* et *G. p. gambiensis* à Folonzo.

Le profil général de l’activité des espèces étudiées dans la réserve de Folonzo montre que *G. tachinoides* et *G. p. gambiensis* ont un rythme d’activité similaire, de type unimodal (figure 4). Les captures chez les deux espèces augmentent progressivement pour atteindre un maximum en milieu de journée – 12 h 00 et 14 h 00, respectivement 19,7 % (n = 2 623) et 22,6 % (n = 472) – avant de décroître aux heures moins chaudes de la fin de journée. Au niveau intraspécifique, la comparaison entre sexes n’indique pas de réelle différence pour les deux espèces. Chez *G. tachinoides*, les femelles atteignent leur activité maximale à 12 h 00 (24 % des femelles) et les mâles à 14 h 00 (22,5 % des mâles), alors que chez *G. p. gambiensis*, les deux sexes présentent les mêmes rythmes d’activité (figure 5).

## Discussion

Nous avons observé que la hauteur de capture d’une même espèce (*G. p. gambiensis*) varie en fonction du contexte environnemental et de la présence d’autres espèces. L’espèce dominante dans le biotope (*G. tachinoides* à Folonzo, et *G. p. gambiensis* à Kartasso) est celle qui est capturée le plus bas, ce qui pourrait être relié avec une stratégie d’économie de ressources énergétiques. Lorsque *G. p. gambiensis*, à Folonzo, est en compétition avec *G. tachinoides*, mais minoritaire, elle est capturée plus haut. Ces différences observées reflètent-elles uniquement des différences dues à l’environnement, ou existe-t-il des phénomènes de compétition ou de différence de comportement de pose sur le leurre ?

### Espèces présentes

Avec le système innovant de capture utilisé (l’écran couvert de film adhésif), permettant de capturer toutes les glossines attirées par le leurre visuel et qui s’y posent, et non pas seulement la proportion qui entrerait dans le système de capture du piège, nous confirmons la prépondérance de *G. tachinoides* dans les galeries forestières conservées de Folonzo sur la Comoé, comme cela avait été observé antérieurement par piégeage (Laveissière *et al.*, 1981 ; Rayaissé *et al.*, 2009). Cependant lors de ces études, la proportion de *G. m. submorsitans* était de loin supérieure à celle de *G. p. gambiensis*, ce qui n’est pas le cas dans l’étude présente. Cela s’explique par le fait que notre étude a été exclusivement menée en galerie forestière, qui n’est pas le biotope de prédilection de *G. m. submorsitans*, abondante plutôt en savane. La présence presque exclusive de *G. p. gambiensis* à Kartasso n’est pas en soit surprenante au vu de la configuration de la végétation bordant la Pindia à ce niveau : cordon guinéen, galerie fermée, même si elle est dégradée. En effet, *G. p. gambiensis* est une glossine “de source”, et la Pindia constitue l’un des affluents du Mouhoun proche des sources, alors que *G. tachinoides* sur le Mouhoun est trouvée plus en aval lorsque la rivière s’élargit et la galerie s’ouvre (Bouyer *et al.*, 2005).

### Distribution verticale

De façon générale, les résultats observés dans notre étude confirment que les glossines du groupe *palpalis* volent bas, puisqu’elles sont capturées à 55,12 cm au dessus du sol pour *G. tachinoides*, et 64,72 cm pour *G. p. gambiensis*. Ce comportement avait été mentionné par Laveissière (1987) chez *G. p. palpalis* en zone de forêt, comme en zone de savane pour *G. tachinoides* (Laveissière, 1976), et est à l’origine du développement des pièges et écrans qui sont toujours placés à une hauteur faible au-dessus du sol. Cette faible hauteur de vol constitue probablement pour la glossine une façon de limiter ses dépenses énergétiques au cours de son déplacement (Bursell *et al.*, 1974). L’individu aurait une capacité d’autant plus grande à s’éloigner du sol qu’il est de grande taille (Taylor, 1974) ; en particulier, au sein d’une espèce, les femelles qui sont de plus grandes, voleraient plus haut que les mâles, comme observé avec les deux espèces à Folonzo.

Au niveau interspécifique, Vale *et al.* (1984) observent au Zimbabwe que *G. pallidipes* a de plus grandes capacités de vol que *G. m. morsitans* (la première étant de taille supérieure à la seconde), ce qui a pour conséquence un impact différent des méthodes de lutte. Rapportée à notre étude et à la hauteur de vol, coûteuse en énergie, on s’attendrait donc à voir *G. tachinoides*, plus petite, voler plus bas que *G. p. gambiensis*. C’est effectivement le cas à Folonzo où les deux espèces cohabitent en sympatrie. Toutefois, c’est à Kartasso, où elle est seule, que *G. p. gambiensis* est capturée le plus bas, ceci sans doute en relation avec la dégradation de la galerie qui laisse entrer les rayons solaires, et crée une atmosphère plus chaude la conduisant à économiser ses ressources énergétiques.

Il faut toutefois avoir conscience que la glossine, à l’approche du leurre, présente un comportement qui va la mener à tourner en cercle autour de ce leurre (Green, 1989 ; Vale, 1993) avant de s’y poser. Ce comportement pourrait donc éventuellement faire varier la hauteur à laquelle elle se pose. Des observations contradictoires sur le comportement de pose sont ainsi rapportées : Nash (1948) pense que *G. p. gambiensis* a tendance à piquer les parties les plus hautes de l’homme, alors que Bouyer *et al.* (2008) rapportent que *G. tachinoides* et *G. p. gambiensis* piquent toutes les deux les parties les plus basses de bovins expérimentalement exposés. Ainsi, ce comportement de pose pourrait varier selon l’hôte, ou le leurre, mais n’explique pas nos observations, puisque dans notre étude, *G. p. gambiensis* est capturée à des hauteurs différentes avec le même leurre, à Kartasso et à Folonzo.

Finalement, il paraîtrait curieux de ne pas souligner que dans les deux situations, c’est l’espèce dominante en termes de densité (*G. p. gambiensis* à Kartasso qui est seule, et *G. tachinoides* à Folonzo) qui est capturée le plus bas. Cette constatation pourrait être interprétée comme un avantage permettant de maximiser l’efficacité dans la recherche des hôtes nourriciers, et minimiser les dépenses énergétiques liées au vol. Une autre observation étaye cette hypothèse de compétition : les graphiques des hauteurs de vol au cours de la journée montrent bien que si les hauteurs spécifiques varient en cours de journée, elles ne se chevauchent pas, donnant l’impression d’une exclusion mutuelle. Ces résultats sont à rapprocher des observations de compétition entre espèces pour le succès de repas de sang sur bovins décrites au Zimbabwé (Torr & Mwangiro, 2000).

On observe aussi que *G. p. gambiensis* réduit sa hauteur de vol au fur et à mesure que la chaleur augmente, ce qui confirme la relation entre chaleur, métabolisme basé sur la proline et ressources énergétiques mentionnée par (Hargrove, 1980) au Zimbabwé. Ces variations dans la journée sont moins prononcées chez *G. tachinoides*, connue pour être plus xérophile.

### Rythme d’activité

*G. p. gambiensis* et *G. tachinoides* présentent un cycle d’activité unimodal avec un pic commun aux environs de 14 h 00, en début de saison des pluies (mois de mai). Chez d’autres espèces et dans d’autres type d’habitats – *G. palpalis* et *G. caligine*a dans les mangroves du Cameroun (Eouzan & Ferrara, 1978) –, une compétition interspécifique avait également été proposée pour interpréter les différences dans les rythmes d’activité journalière.

Le léger décalage des pics d’activités (12 h 00 contre 14 h 00) des mâles et femelles de *G. tachinoides* à Folonzo a été aussi observé chez d’autres espèces, par fois même de manière plus importante (Carnevale & Adam, 1971 ; Elsen, 1973 ; Challier, 1973). Sa modestie ici pourrait s’expliquer par l’état de conservation de la galerie qui limite les variations de températures, la végétation maintenant la température et l’hygrométrie presque constantes et favorables à l’activité des glossines. Les pics d’activité sont ainsi atteints à la période la plus chaude de la journée, qui correspond à la présence des animaux sauvages venus alors de la savane pour s’abreuver et prendre un bain au niveau du cours d’eau.

## Conclusion

Grâce à un système de capture qui permet de retenir toutes les glossines posées sur le leurre, nous avons montré qu’il existe des différences de hauteur de capture entre deux espèces vivant en sympatrie, et entre populations d’une même espèce dans des localités distinctes. Outre la nature de la galerie forestière, ces interactions pourraient être imputées à une compétition basée sur les ressources énergétiques, et/ou sur des comportements plus locaux, comme le comportement de pose, correspondant à l’attaque d’un hôte potentiel. Des études complémentaires permettront d’aller plus loin dans la compréhension de ces interactions. En effet, ces différences pourraient expliquer des différences d’efficacité de méthodes de lutte basées sur des systèmes attractifs. L’on pense en particulier aux “traînées” d’odeur qui pourraient plus affecter une espèce qu’une autre selon leur hauteur de vol, ou l’efficacité de certains types de pièges qui pourrait également varier si ces phénomènes de compétition par exclusion existent de manière importante.
